# Analysis of Toxic Amyloid Fibril Interactions at Natively Derived Membranes by Ellipsometry

**DOI:** 10.1371/journal.pone.0132309

**Published:** 2015-07-14

**Authors:** Rachel A. S. Smith, Aleksey Nabok, Ben J. F. Blakeman, Wei-Feng Xue, Benjamin Abell, David P. Smith

**Affiliations:** 1 Biomedical Research Centre, Sheffield Hallam University, Sheffield, United Kingdom; 2 Materials and Engineering Research Institute, Sheffield Hallam University, Sheffield, United Kingdom; 3 School of Biosciences, University of Kent, Canterbury, Kent, United Kingdom; University of Waterloo, CANADA

## Abstract

There is an ongoing debate regarding the culprits of cytotoxicity associated with amyloid disorders. Although small pre-fibrillar amyloid oligomers have been implicated as the primary toxic species, the fibrillar amyloid material itself can also induce cytotoxicity. To investigate membrane disruption and cytotoxic effects associated with intact and fragmented fibrils, the novel *in situ* spectroscopic technique of Total Internal Reflection Ellipsometry (TIRE) was used. Fibril lipid interactions were monitored using natively derived whole cell membranes as a model of the *in vivo* environment. We show that fragmented fibrils have an increased ability to disrupt these natively derived membranes by causing a loss of material from the deposited surface when compared with unfragmented fibrils. This effect was corroborated by observations of membrane disruption in live cells, and by dye release assay using synthetic liposomes. Through these studies we demonstrate the use of TIRE for the analysis of protein-lipid interactions on natively derived lipid surfaces, and provide an explanation on how amyloid fibrils can cause a toxic gain of function, while entangled amyloid plaques exert minimal biological activity.

## Introduction

Protein misfolding and aggregation are implicated in numerous neurodegenerative diseases, such as Alzheimer's disease and Parkinson's disease. These diseases are characterised by the deposition of both intra- and extracellular amyloid deposits [1]. Amyloid fibrils originate from proteins or peptides that self-assemble through a nucleation dependent mechanism to form fibrillar aggregates rich in β-sheet content. These fibrils are long, unbranched polymers, typically 10 nm in diameter, and can be up to several micrometres long [2–3]. Amyloid material has characteristic properties such as the ability to bind the benzothiazole dye thioflavin-T, and the conformation-specific LOC antibody [4]. It is hypothesised that all proteins have the ability to misfold and aggregate into amyloid and the fibril structure for a diverse range of proteins is known to be remarkably similar [5]. To date, more than 25 amyloid forming proteins have been identified in disease-associated amyloid deposits, including α-synuclein (α-syn) in Parkinson’s Disease (PD), amyloid-β peptide (Aβ) in Alzheimer’s disease (AD), and prion protein (PrP) in transmissible spongiform encephalopathies [6]. Furthermore, aggregation rates can be increased as a result of genetic mutations associated with early onset familial forms of amyloid-associated diseases, such as the point mutations A53T, A30P and E46K found with in α-syn in early onset PD [7–10].

Identifying the culprits of the cytotoxicity associated with amyloid is complex due to the heterogeneity of amyloid assembly and the large numbers of different species identified during amyloid formation [11]. During fibril formation, a range of oligomeric species may accumulate, thereby making it difficult to identify a sole culprit of cytotoxicity. Studies have focused on the small prefibrillar oligomers as active toxic species [12–14] and in some cases, it is thought that pre-fibrillar oligomers are implicated as the primary source of toxicity via the formation of membrane pores, membrane thinning and destabilization [2,15]. Recently, there has been an increased focus upon the physical attributes of amyloid fibrils, and there are increasing examples of fibrils associated with cytotoxicity [3, 16–17]. Due to the varied biological responses elicited by amyloid fibrils of different size and lengths, it has been proposed that the cytotoxic effects they are associated with may not only depend on their chemical composition or molecular properties but their physical attributes such as length, width and surface area [3, 18–20]. Using three different proteins (human β_2_-microglobulin (β_2_m), lysozyme and α-syn) as models of amyloid fibrils associated with disease, it has been demonstrated that fragmentation of fibrils had a marked effect on amyloid cytotoxicity. Fragmented fibrils from all three proteins showed increased disruption of liposome membranes and reduced cell viability indicated using MTT assays, when compared with unfragmented but otherwise identical samples. This suggests that the reduction of fibril length presents a mechanism by which fibril associated cytotoxicity could be enhanced [17]. Fragmentation was achieved by stirring, resulting in a reduction of fibril length and an increase of the total number of fibrillar particles, whilst maintaining the overall fibril load. Fragmentation leads to a decrease in the surface area to the long axis of the individual fibril and an increase in the total surface area of fibril ends. Subsequently, there is an increase in biological availability as the number of fibril ends rise. Fibril ends are reactive entities acting as sites for monomer addition during fibril elongation [21]. A rise in fibril ends may increase the fibrils ability to directly bind and disrupt membranes causing enhanced cytotoxic effects. This has been corroborated by numerous other studies, including a study that used β_2_m fibrils and showed enhanced interaction of fragmented fibrils with liposomes causing membrane disruption, particularly noted in proximity to fibril ends [22]. These studies suggest a mechanism by which fibrils may cause membrane disruption leading to the cellular dysfunction associated with many amyloid diseases. However, these biophysical investigations have used large unilamellar vesicles (LUVs) as a model system, instead of native cellular membranes.

Here, we use the biophysical technique of total internal reflection elliposometry (TIRE) to characterise the interactions of α-syn, Aβ_40_ and hen egg white lysozyme fibrils with natively derived whole cells (SH-SY5Y human neuroblastoma cell line) monitoring the protein-lipid interactions *in-situ*. TIRE is a non-destructive and label free optical method measuring changes in the polarization of reflected light; it is extremely sensitive to changes in optical parameters of the reflecting substrate including values of thickness. We have recently developed TIRE for diverse biological applications, and have found that it can be 10 times more sensitive than conventional surface plasmon resonance (SPR) [23]. Our methodology allows the deposition of whole organelles or cells onto a gold coated surface, resulting in a natively derived membrane on which we can perform interaction studies. Previously, we used this method to study protein receptor interactions on both chloroplasts [24] and plasma membranes [25], indicating that the deposited membrane remains largely intact and is capable of binding to soluble proteins in a specific manner. Here we analyze unfragmented and fragmented amyloid fibril interactions with natively derived cellular membranes. We demonstrate a correlation between fibril length and the ability to bind to the native membrane. Our data indicate that fragmented fibrils cause increased membrane disruption and loss of deposited membrane material when compared to unfragmented fibrils.

## Materials and Methods

### Expression and purification of recombinant wild type α-syn

Human, recombinant wild type α-syn was expressed in *E*. *Coli* BL21 (DE3) and purified as described previously [26]. Briefly α-syn was expressed in *E*. *coli* BL21(DE3) cells (New England Biolabs, Ipswich, MA). Expression was induced by IPTG (100 mM) at 37°C for 3 hours. Bacterial cells were harvested by centrifugation at 20°C, 10,000 rpm and resuspended in lysis buffer (10 mM Tris-HCl (pH 8.0), 100 μg/mL lysozyme, 1 mM phenylmethylsulphonyl flouride (PMSF), 20 μg/mL DNase, 20 μg/mL Rnase). The protein lysate was subjected to anion exchange followed by size exclusion chromatography. Lyophilised purified α-syn was stored at -20°C. Protein concentration was measured using an UV/Vis spectrophotometer (Jenway 6715) at absorbance 280 nm with an extinction coefficient of 5960 M^-1^cm^-1^.

### Preparation amyloid fibrils

α-Syn fibrils were prepared by dissolving lyophilised α-syn protein to a final concentration of 1 mg/mL in 25 mM sodium phosphate buffer (pH 7.4), and incubating on an orbital shaker at 37°C, 300 rpm for 7 days. Quiescent Aβ_40_ fibrils were prepared by dissolving 1 mg of lyophilised peptide (Bachem, Bubendorf, Switzerland) without further purification in 200 μL hexafluoroisopropanol (HFIP) and vacuum dried for 24 hours for HFIP removal. The peptide film was re-suspended in 20 mM sodium hydroxide (NaOH), milliQ water, 10x PBS in a 2:7:1 ratio to give a final concentration of 1 mg/mL and then incubated at 37°C for 7 days. Lysozyme (Sigma Aldrich) fibrils were prepared by dissolving 1 mg of lyophilised protein in 1 mL 0.1 M HCl (pH 1.6) and incubated overnight at 65°C with agitation, 550 rpm. Fragmented fibrils were formed by freeze-thawing (-20°C) each sample, followed by 30 min sonication (Branson 1210 sonicator).

### AFM imaging of amyloid fibrils

Fibril samples were imaged by Atomic force microscopy using a Bruker Multimode 8 scanning probe microscope (Bruker) operating under peak-force tapping mode. The AFM specimens were prepared by incubating a freshly cleaved mica surface with 20 μL fibril samples. The fibril samples were made by diluting stock solutions with filter sterilized de-ionized water to final protein monomer-equivalent concentration of 0.2 mg/mL (α-syn), 0.1 mg/mL (lysozyme), or 0.5 mg/mL (Aβ_40_) and were incubated on the mica surfaces for 5 minutes (α-syn and lysozyme) or 1 hour (Aβ_40_). The samples were subsequently washed with 1 mL of filter sterilized de-ionized water, before drying with filter paper followed by a gentle stream of nitrogen gas. Images of ~10x10 nm/pixel (e.g. 10x10 μm, 1024 x 1024 pixels) or higher resolutions were acquired. All images were processed using supplied software (NanoScope Analysis version 1.3 or 1.4, Bruker) to remove sample tilt and scanner bow.

### Dot Blotting

Nitrocellulose membranes (0.45 μm, GE Healthcare, Sweden) were spotted with 1μL of monomeric or fibrillar species at 1 mg/mL (α-syn, Aβ_40_ and lysozyme) as prepared above alongside bovine serum albumin (BSA) as a negative control. The membrane was blocked with 5% milk in Tris buffer (pH 8.0) containing 0.1% Tween 20. Primary antibody against amyloid fibril LOC (Millipore, UK) (fibril specific antibody) was diluted in TBS-T (2% milk) at 1:1000 and incubated with membrane for 1 hour. Three wash steps were performed for 10 minutes with TBS-T. Secondary antibody IRDye 800CW goat anti-rabbit IgG (H+L) (Li-Cor) was applied at 1:20000 dilution and incubated at room temperature for 1 hour. Visualization was performed using the Li-Cor Odyssey and Odyssey Infrared Imaging software at wavelength 800 nm.

### Liposome Dye Release Assay

Large unilamellar vesicles (LUV) were prepared as previously described [17]. Briefly LUVs were made using a lipid mixture of 1.6 mg/mL phosphatidylcholine and 0.4 mg/mL phosphatidylserine purified from chicken egg (Avanti lipids) to give 20% negatively charged head groups. The lipids were dissolved in 1 mL (100%) Chloroform followed by evaporation under a stream of Nitrogen gas. The dried lipid film was re-suspended in 50 mM carboxyfluorescein in 50 mM sodium phosphate, 10 mM sodium chloride and 1 mM EDTA (pH7.4) (Sigma) and subjected to extrusion through a polycarbonate filter with 0.1 μm pore size. The carboxyfluorescein encapsulated LUVs were washed 3 times in liposome buffer (50 mM sodium phosphate, 107 mM sodium chloride and 1 mM EDTA pH 7.4), pelleted by ultracentrifugation (Beckman Optima TLX) at 100,000 x g for twenty minutes, resuspended in liposome buffer, and used within 48 hours. Dye release assay reactions consisted of 2 μL of LUV stock solution diluted with 188 μL liposome buffer, and 10 μL fibril sample (α-syn 100 μg/mL, Aβ_40_ 20 μg/mL and lysozyme 20 μg/mL) to give a final volume of 200 μL. Fluorescence emission of carboxyfluorescein was quantified at 520 nm with an excitation wavelength of 480 nm, using Tecan Infinite plate reader. Controls using LUV with liposome buffer only and addition of 20% v/v Triton X-100 were also included for every reaction. Statistical significance between unfragmented and fragmented dye release was determined by a Mann Whitney U test P ≤ 0.05 (*) or P ≤ 0.005 (**).

### SH-SY5Y Cell Line Culture

All cell culture methods were carried out under sterile conditions in a class II laminar flow cabinet. The SH-SY5Y neuroblastoma cells were obtained from the European Collection of Cell Cultures. Thawed stocks of adherent cells were routinely grown and passaged in a cell culture incubator at 37°C and 5% CO_2_ in 75 cm^2^ cell culture flasks with 20 mL of DMEM (Dulbecco’s Modified Eagle’s Medium supplemented with 2% (v/v) L-alanyl-L-glutamine (GIBCOH GlutaMAX) and 2% penicillin-streptomycin (Invitrogen). Cell monolayers were determined to be near-confluent at approximately 80%. The cells were detached from the flask by incubating in 3 mL of trypsin/EDTA solution (0.25% trypsin, 0.02% EDTA) at 37°C for 5 minutes. Trypsin digestion was stopped by addition of 7 mL of supplemented DMEM. The resulting cell solution was placed into 50 mL Falcon tubes and centrifuged for 5 minutes at 1000 rpm and the supernatant discarded. Cell pellets were washed three times by re-suspension and centrifugation in 10 mL of 1x phosphate buffered saline (PBS). The pellet was re-suspended in 1x PBS, aliquotted, snap-frozen in liquid nitrogen and stored at -80°C.

### CellTox Green Membrane integrity Assay

Following manufacturer guidelines the Express, “No-Step Addition at Seeding” method (Promega) was performed using the human neuronal cell line SH-SY5Y. Briefly, 10 μL of celltox dye was added per 5 mL cell culture. Cells were plated at a density of 2x10^4^ cells/well in phenol free media with 10% FCS, into sterile 96 wellplates (50 μL) and allowed to adhere overnight at 37°C with 5% CO_2_. The test compounds were applied at a 10% v/v giving final concentrations of 0.1 mg/mL α-syn, Aβ_40_ and lysozyme. A volume-matched negative control (CDMEM), and a positive control for primary necrosis using the kits lysis solution at 1:25 ratio were also applied. Fluorescence was measured using a Tecan Infinite plate reader (excitation 485 nm and emission 520 nm). The experiment was performed over a 72 hour period, with readings every 24 hours. Statistical significance between unfragmented and fragmented toxicity at each time point was determined by a Mann Whitney U test P ≤ 0.05 (*) or P ≤ 0.005 (**).

### Langmuir-Schaefer & TIRE measurements

Cell deposition and TIRE analysis were performed as previously described [24–25]. Briefly, Langmuir-Schaefer deposition was performed using standard microscopic glass sides (1" x 1") coated with chromium (Cr 3nm thick) and gold (Au 25 nm thick) via thermal evaporation (Edwards Life Sciences A360 metallizing unit, Irvine, CA). The metal coated slides were incubated overnight at 4°C in a 0.1 M cysteamine-Hydrochloride (Cys-HCl) solution to achieve positively charged slides. A Langmuir-trough (Nima PS4) was used for the assembly of membrane layers (SH-SY5Y cells), on the trough’s surface of de-ionized water. A target pressure of 20 mN/m was used for deposition and the Langmuir-Schaefer method of horizontal lifting [27] was utilized for transferring membranes onto the glass slides, which was monitored by the recording of surface pressure (π). Protein samples were sequentially injected into the TIRE cell (200 μL) over an increasing concentration range, and incubated for 30 minutes per concentration point. TIRE measurements and curve fitting were performed using the J.A Woollam spectroscopic ellipsometer M2000 with a rotating compensator, operating in the spectral range 370–1000 nm. During sample incubation, two types of ellipsometric measurements were performed: (1) dynamic TIRE spectral measurements where multiple TIRE spectra were recorded during protein adsorption; and (2) TIRE single spectrum scan recording after completion of every adsorption step using standard buffer solution (1 x PBS, pH 8.0). Software provided by J.A Woollam Ltd enabled the modelling of the reflection system and subsequent evaluation of the thickness and refractive index of adsorbed molecular layers, comparing experimental and theoretical values of Ψ and Δ (previously described [24]). A single spectroscopic scan performed after completion of sample incubation per concentration enabled the recording and interpretation of direct changes to membrane thickness (Δd, nm). The Δd (nm) was tested for normality using the Shapiro Wilk test and statistical analysis of the changes in surface height was performed using a Kruskal Wallis test with a Conover-Inman post hoc-test.

## Results

**Fig 1:**
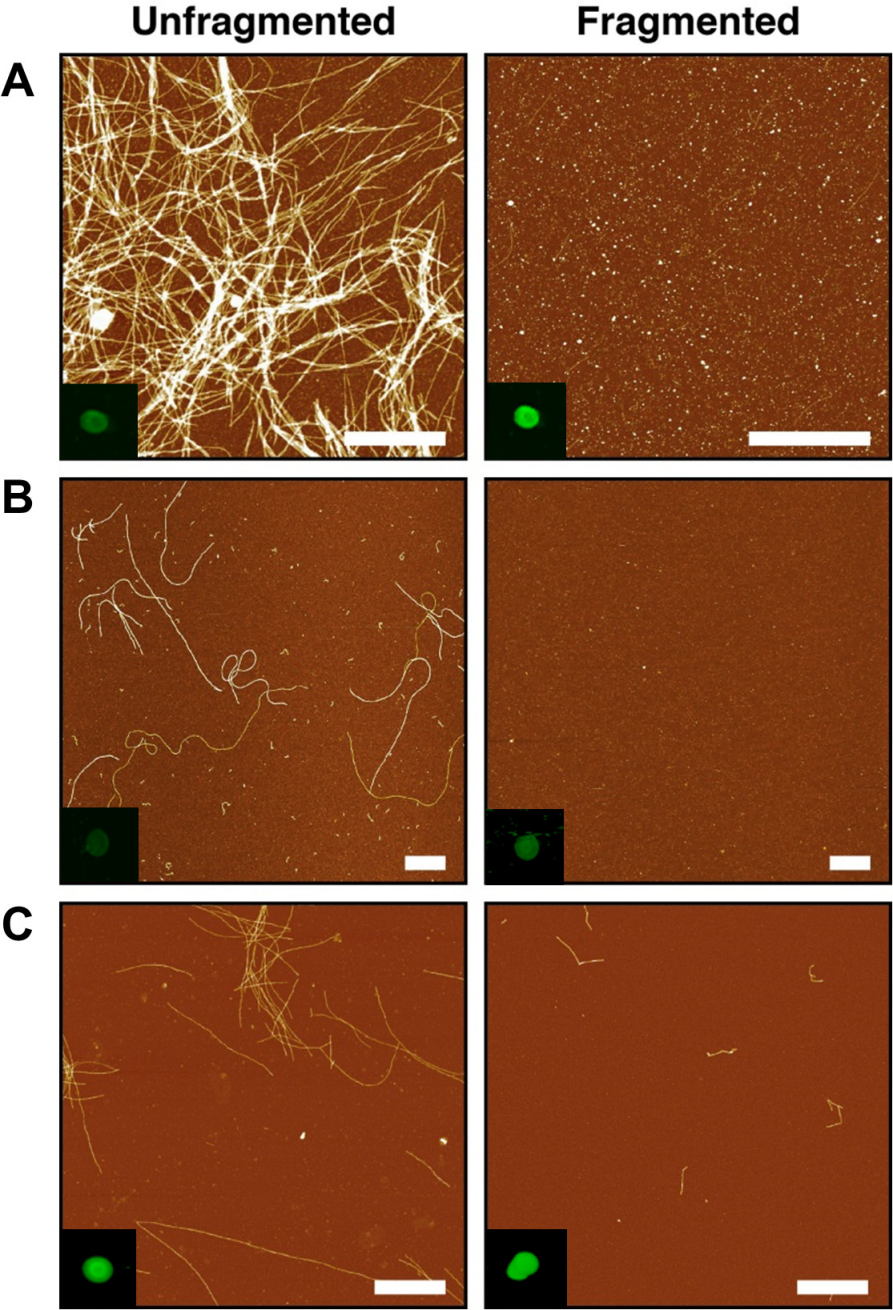
Amyloid fibril characterization. AFM height images of fibril samples adhered to mica surface (scale bar equals 1 μm) (A) α-syn, (B) Lysozyme and (C) Aβ_40_ with dot blot analysis of fibril samples (inset) by anti-fibril LOC antibody specific to generic epitopes common in amyloid fibrils.

To investigate the nature of fibril-membrane interactions, amyloid fibrils were produced following established methods for α-syn [17], Aβ_40_ [28] and lysozyme [29]. Protein samples of identical starting material were aliquotted, and short, fragmented fibrils were achieved through freezing and sonication. Fibril samples formed from all three proteins, both unfragmented and fragmented, were imaged using AFM. This confirmed that the dimensions and morphological features were typical of the unbranched amyloid polymers previously documented [17] with each sample also demonstrating increased ThT fluorescence (data not shown), representative of increased β-pleated sheet structures characteristic of amyloid fibrils (Fig 1A–1C). Coverage of fibrils onto the mica varies depend on fibril length as has been observed previously [17, 30] and is expected to change for the fragmented samples compared with their unfragmented counterparts. Fibrillar material within samples was also tested by antibody using the LOC anti-amyloid fibril antibody (Millipore UK), which recognises generic epitopes common to amyloid fibrils. A negative control using BSA showed no immunoreactivity, whilst binding could be observed for both unfragmented and fragmented fibrils, confirming the presence of amyloid in all cases (Fig 1 inset).

### Effect of liposome membrane disruption by fibrils

**Fig 2:**
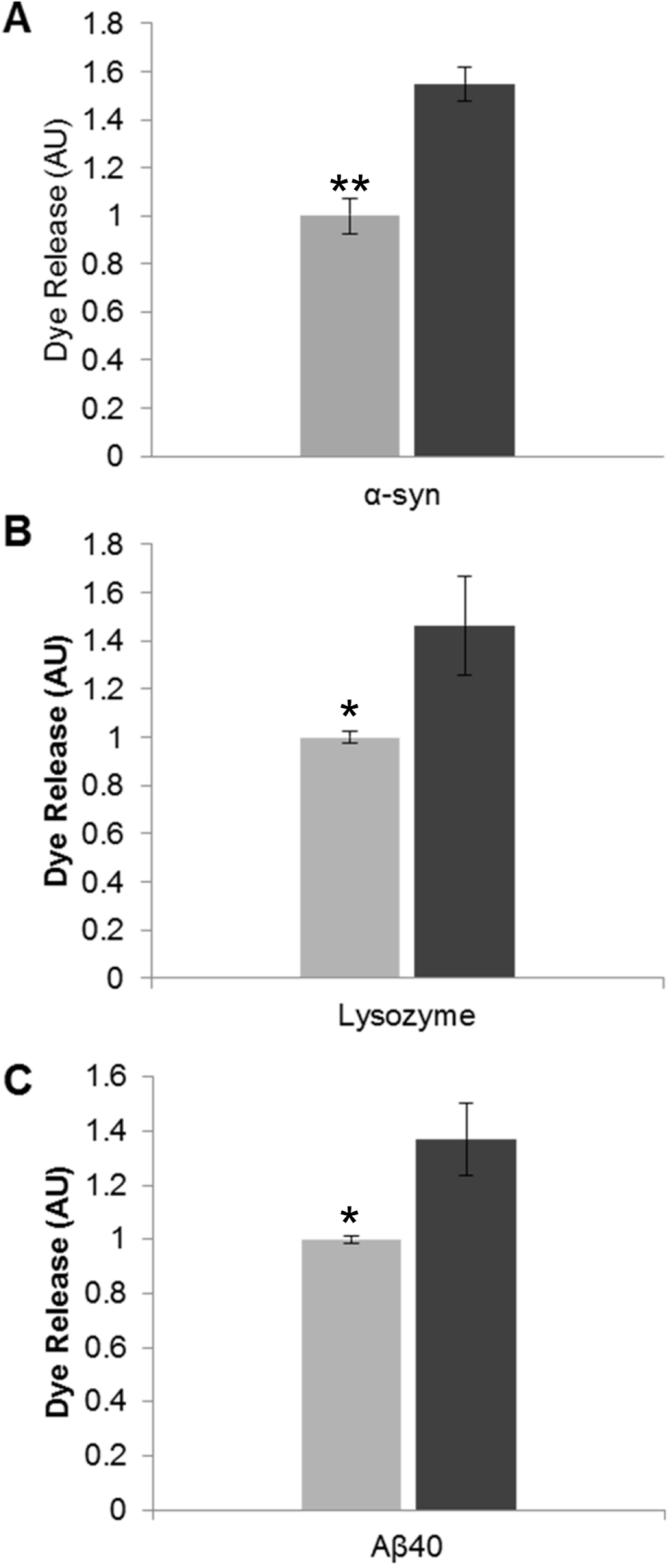
The effect of fibril fragmentation on length and its capacity to cause membrane disruption. Liposome dye release assay using a model lipid membrane formed from 80% (w/w) phosphatidylcholine and 20% (w/w) phosphatidylserine encapsulating the fluorescent probe carboxyfluorescein. Dye-encapsulated vesicles were incubated with amyloid fibril samples (30 min): (A) α-Syn 100 μg/mL (B) Lysozyme 20 μg/mL and (C) Aβ40 20 μg/mL. Fluorescence was then recorded and the fold increase in dye release was normalised against unfragmented fibril fluorescence. Light grey bars denote unfragmented fibril samples and dark grey bars denote fragmented fibril samples (n = 3, error bars show SE). Statistical significance between the unfragmented and fragmented fibril dye release response was determined by a Mann Whitney U test P ≤ 0.05 (*) or P ≤ 0.005 (**).

It has been shown that fibril-membrane interactions cause membrane disruption [22, 31] and in particular that fragmented fibrils have enhanced membrane disruption capability compared to their longer, unfragmented counterparts [17]. In order to confirm that the fibrils produced acted in the same manner as those published previously, we monitored the ability of both fibril samples to disrupt synthetic membranes. Dye-release assays were performed using LUVs formed from 80% phosphatidylcholine and 20% phosphatidylserine to reflect the physiological lipid composition and charge of the plasma membrane [32], and encapsulated the self-quenching fluorescent dye carboxyfluorescein at pH 7.4. Successful dye encapsulation in LUVs was demonstrated by the ability to trigger dye release with the addition of Triton X-100 (20% v/v), which is known to dissolve membranes. The change in signal upon addition of fibrils to the dye encapsulated LUVs was recorded. The degree of dye release was normalised against unfragmented fibril samples, and showed a ~0.5 fold increase for all three fragmented amyloid proteins compared to their unfragmented counterparts. This reflects an enhanced ability of fragmented fibrils to disrupt dye-encapsulated liposomes (Fig 2).

### Reduced cell membrane integrity in the presence of fragmented fibrils

**Fig 3:**
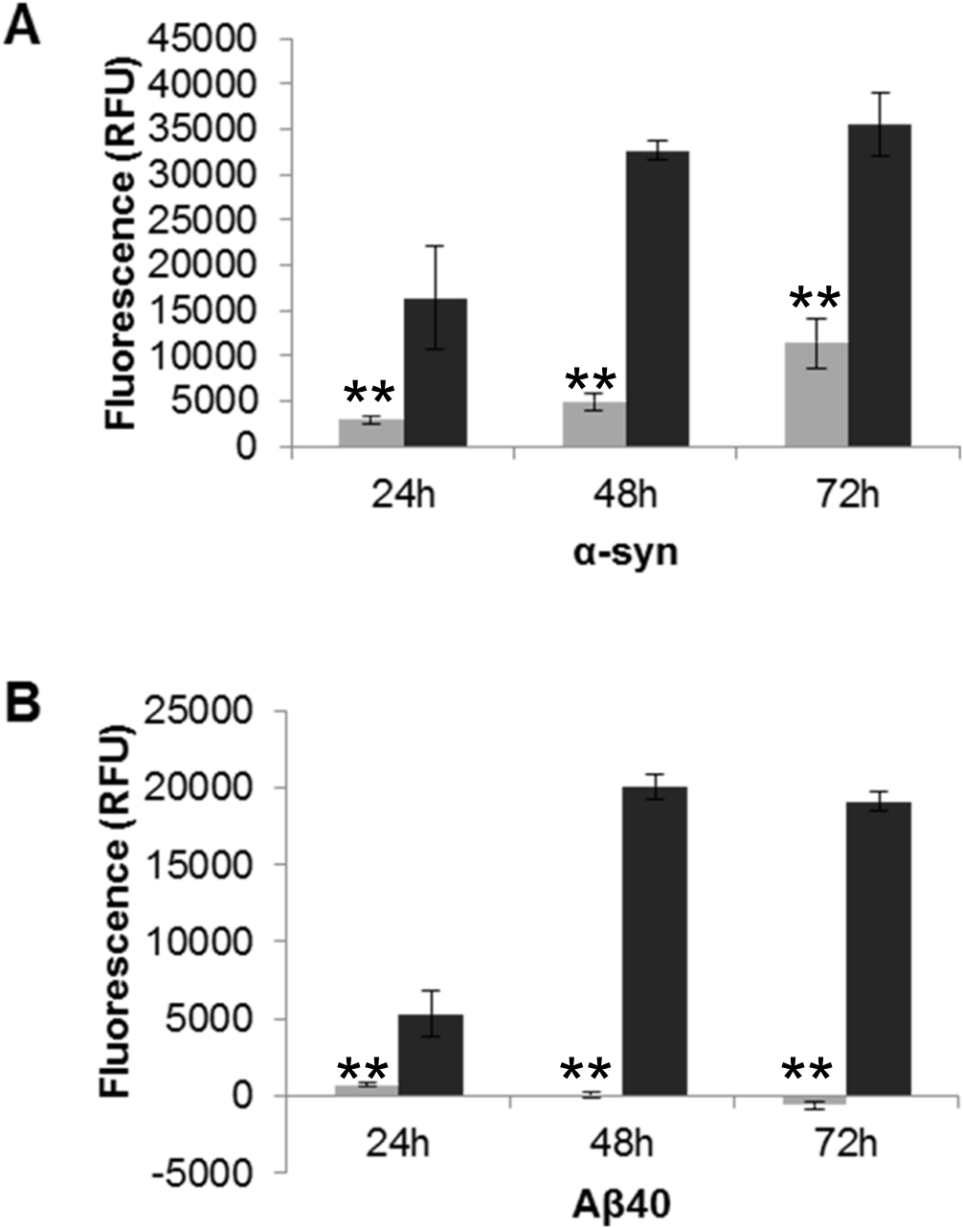
The effect of fragmented fibril on cell viability by cell membrane integrity assay. SH-SY5Y cells (2x10^4^/well) were plated with the addition of the CellTox dye and allowed to adhere. The unfragmented and fragmented fibril samples were then added (A) α-syn 7 μM and (B) Aβ_40_ 10 μM. Fluorescence was recorded over a period of 72 hours (520_Em_/ 485_Ex_). Light grey bars denote unfragmented fibril samples and dark grey bars denote fragmented fibril samples (n = 3, error bars show SE). Unfragmented (light grey) and fragmented fibril samples (dark grey) (n = 6, SE). Statistical significance between toxicity of unfragmented and fragmented fibrils at each time point was determined by a Mann Whitney U test P ≤ 0.05 (*) or P ≤ 0.005 (**).

To determine whether the enhanced membrane disruption by fragmented fibril samples is replicated in living cells, the human neuroblastoma cell line SH-SY5Y was incubated with both fragmented and unfragmented fibril samples for each protein. Cell membrane integrity was measured over 72 hours using the CellTox Green cytotoxicity Assay (Promega), which measures changes in membrane integrity by binding of the cyanine dye to released DNA. Fig 3 shows that treatment with fragmented fibril samples from α-syn and Aβ_40_ caused higher fluorescence intensity than observed for unfragmented fibrils, reflecting loss of membrane integrity and increased cytotoxicity. The fragmented fibrils from α-syn and Aβ_40_ both exhibit cytotoxic effects within 24 hours, with fluorescence intensity nearly three times higher than incubation with unfragmented fibril samples (Fig 3). Lysozyme fibrils were prepared in pH 1.6 buffer and in this case the solvent control proved toxic to the cells, so it was not possible to determine its effect on live cells. This live cell membrane integrity based assay shows the increased fibril-membrane interaction by fragmentation of fibrils, which is consistent with the findings for synthetic vesicles.

### Monitoring fibril-membrane interactions *in-situ* using total internal reflection elliposometry (TIRE)

TIRE has recently been adapted to enable the study of protein and peptide interactions at natively derived cellular membranes, utilising electrostatic deposition of cells using the Langmuir-Schaefer method. Here we have used TIRE to monitor *in-situ* protein binding of amyloid fibril preparations, monitoring changes in thickness (Δ*d*) of a natively derived plasma membrane to study the effects of fibril-membrane interactions. We used Langmuir-Schaefer deposition methods to create a natively derived membrane surface originating from the whole neuronal cell deposition of SH-SY5Y cells. The cells are found to be relatively intact with no membrane fragmentation after the freeze-thaw process confirming membrane integrity using a light microscope. Furthermore, using confocal microscopy the deposited membrane was imaged documenting a homogenous layer of cells saturating the glass slide [25].

A limitation of ellipsometry and SPR is that the simultaneous evaluation of thickness (*d*) and refractive index (*n*) of thin dielectric films (<10 nm) is not possible, and either *d* or *n* must remain fixed during data analysis. The refractive index (*n*) for the deposited organic layers using TIRE was fixed as previously described [33] utilizing the Cauchy dispersion model [34] for all adsorbed layers yielding a value of refractive index *n* = 1.42 (at 633 nm). Similar values have been reported for organic substances varying between 1.44–1.49 [35–36]. Therefore, any changes at the adsorbed molecular layer are associated with direct changes in thickness (*d*). Thus, TIRE allows the monitoring of the average change in thickness of material from a membrane surface as fibrils bind to it. Unfragmented and fragmented fibril samples were injected over the membrane surface, and the membrane thickness measured as the protein monomer equivalent concentrations was increased incrementally.

**Fig 4:**
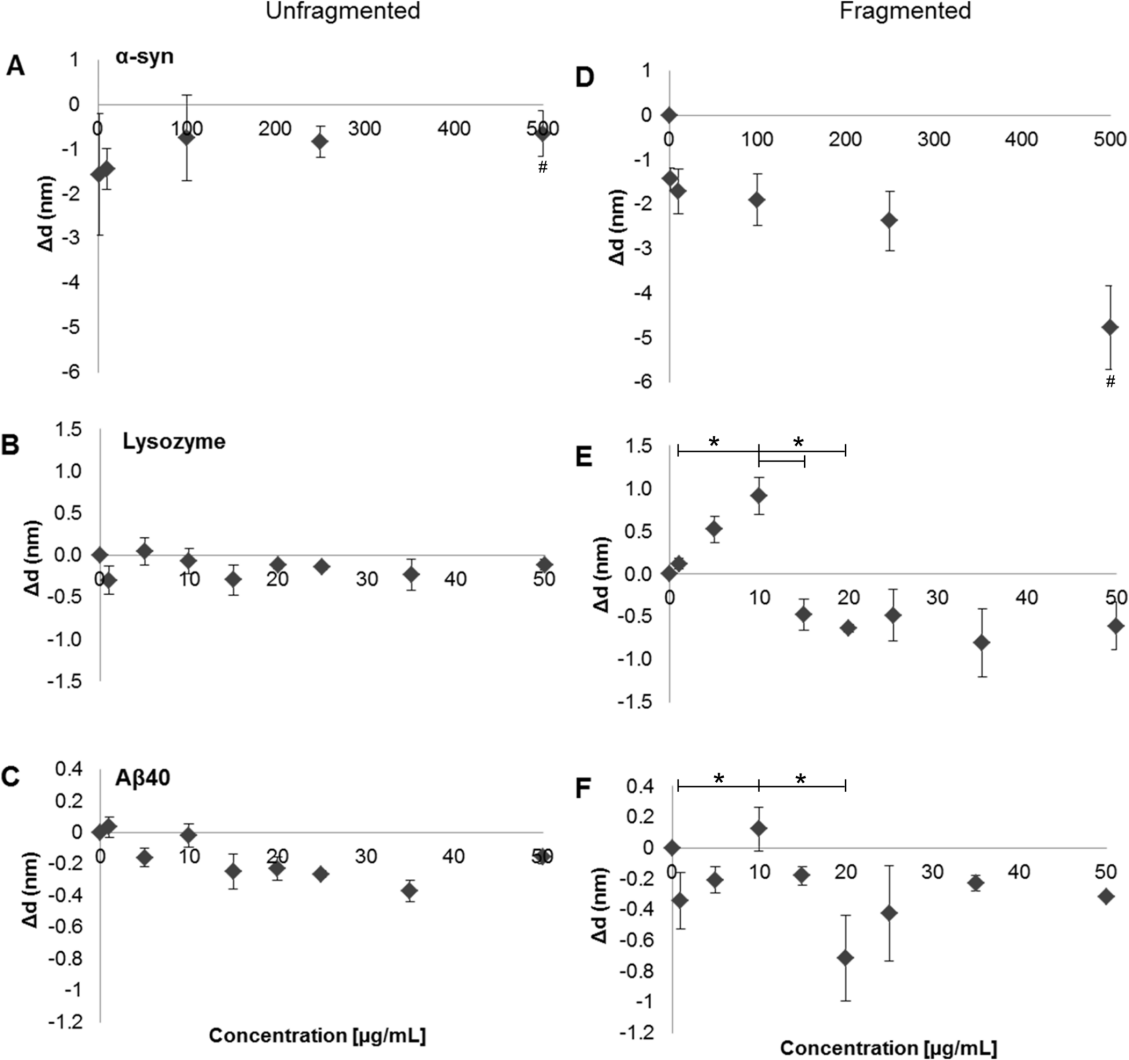
TIRE measurements of membrane disruption at native lipid membrane by the incremental addition of amyloid fibrils. Natively derived cells from the neuronal cell line; SH-SY5Y was deposited by Langmuir-Schaefer deposition and protein: lipid interactions were probed between amyloid fibril samples and plasma membranes. Left plots (A, C and E) show experiments with unfragmented samples and right plots (B, D and F) fragmented samples. Top row plots (A+D) show experiments with α-Syn, middle row (B+E) Lysozyme, and bottom row (C+F) Aβ40. Membrane thickness were measured by change in Δd (nm), which is ploted versus fibril concentration (n = 3, error bars show SE). Statistical significance for the differences in thickness within an experiment on exposure to fragmented fibril samples was determined by Kruskal Wallis test with an Conover-Inman post hoc-test. (*) or (**) denote P ≤ 0.05 and P ≤ 0.005, respectively. Statistical significance between separate unfragmented and fragmented experiments in the case of α-syn was also determined by Kruskal Wallis with an Inman post hoc-test with P ≤ 0.05 (^#^).

A significant loss of material at the TIRE surface of between -0.5 to -1.8 nm can be observed following the addition of fibril samples prepared from all three proteins as the concentration is incrementally increased (Fig 4). Unfragmented α-syn fibril samples initially caused a loss of material (-1.8 nm), which then recovers to ~0.3 nm at 100 μg/mL (Fig 4A), after which there is little change and a plateau at -0.5 nm is observed. α-Syn fragmented fibril counterparts (Fig 4D) demonstrated a continual loss of deposited material after each injection. Unfragmented lysozyme (Fig 4B) and Aβ_40_ (Fig 4C) show reduced membrane disrupting effects compared with α-Syn, causing a minor loss of up to -0.5 nm (Fig 4B & 4C). The data show that at low initial concentrations unfragmented fibrils are disrupting the membrane (Fig 4A–4C). However, as the total concentration of the fibrils increases there is some recovery in the total thickness, presumably as the unfragmented fibrils accumulate on the surface. This accumulation could occur via aggregative fibril-fibril interactions, which limit their membrane interaction, or by the saturation of available lipid binding sites, with a plateau in the fibril-membrane interactions (Fig 4A–4C) as all available binding sites are filled.

Fragmented fibril samples display an increased ability to bind and disrupt natively derived membranes when compared to unfragmented fibril samples. These samples contain fragmented fibrils which are shorter in length and have an increased number of fibril ends when compared to unfragmented fibril samples, as observed by AFM (Fig 1). Initial low concentrations between 1–10 μg/mL for fragmented fibrils of α-syn (Fig 4D) result in a loss of material at the membrane (-1.5 to -2 nm), which is indicative of their cytotoxic effects. When comparing the 500 μg/mL of the unfragmented and fragmented α-syn fibrils, the amount of material lost was significant greater in the fragmented samples (P ≤ 0.05). Lysozyme fragmented fibrils exhibited an initial increase in membrane thickness between 1–10 μg/mL, reaching ~0.9 nm (Fig 4E), whereas Aβ_40_ displays a loss of material at 1 μg/mL of ~0.3 nm thickness followed by a slight gain in thickness between 5–10 μg/mL (Fig 4F). There is a significant increase in material in the case of the fragmented fibrils in both the Lysozyme and Aβ_40_ samples between 1 μg/mL and 10 μg/mL (P ≤ 0.05). However, after the addition of > 10 μg/mL both fragmented Lysozyme and Aβ_40_ fibrils show a noticeable loss of material, a decrease of ~ 1.0 nm in thickness. The drop in material on the surface between 10 μg/ml and 20 μg/mL of added fragmented fibrils for both Lysozyme and Aβ_40_ fibrils was significant (P ≤ 0.05). These observations demonstrate an accumulation of fragmented fibrillar material at the membranes surface, similar to that seen by their unfragmented counterparts. Upon reaching a certain threshold the accumulation of fragmented fibrils results in a loss of material from the surface, presumably via membrane disruption even though additional fibrillar material is being added. The concentration range over which this effect of increasing then decreasing thickness is observed is protein and fibril specific, but the pattern of activity is common to all types studied. Fragmented α-syn fibril samples display a dose dependent effect (Fig 4D) causing a substantial loss of membrane thickness of up to -5 nm at 500 μg/mL, lysozyme fibril samples reach around -1.0 nm at 35 μg/mL (Fig 4E) which remains around this value for the remainder of the concentration range tested, whilst Aβ_40_ causes its greatest loss of membrane at 20 μg/mL (-0.8 nm) (Fig 4F). Overall, analysis from all three proteins using TIRE showed enhanced membrane disruption by fragmented fibrils when compared to their unfragmented counterparts. The enhanced fibril-membrane association of the fragmented fibrils correlates with their ability to induce increased lipid dye release from synthetic LUVs (Fig 2) and disruption of membranes in cell viability assays (Fig 3).

## Discussion

Here we have characterised the interaction of both unfragmented and fragmented amyloid fibril samples with a natively derived membrane, and we show a correlation between membrane interaction and cytotoxicity via membrane disruption. This interaction is dependent on the length of the fibrils in question, with an increase in fibril end number resulting in increased disruption and loss of natively derived lipid material from whole SH-SY5Y cells in an *in vivo* model. Our findings are consistent with those previously described [17], which demonstrated using *in vitro* synthetic lipids that fibril length correlates with their ability to disrupt membranes and consequently reduces cell viability. FTIR spectroscopy has confirmed that no significant structural perturbations occurred upon generation of fragmented fibrils, and the only the physical attribute altered being length. This observation is in accord with our own AFM images (Fig 1) that show a reduction in length of amyloid fibrils after freezing and sonication. The extent of fibril formation and gross morphology was protein specific. However, the generic fibril epitope recognised by the LOC anti-amyloid antibody was present in all three aggregated proteins. The individual differences in protein load causes variances in levels of antibody binding (Fig 1) and subsequent membrane interactions (Fig 4). These observations support the idea that the relationship between primary structure and its propensity to form fibrils is driven by the interactions between polypeptide chains, whereby amino acid sequence can govern the rate of protein aggregation, affecting structural details of grown fibrils [37–39]. However, it is clear in all cases that amyloid material is present, but with differences in total load and morphology.

LUV dye release assays (Fig 2) showed that fibril fragmentation enhanced the ability to disrupt liposome membranes over unfragmented fibrils, which has been previously shown [17]. Hence, the results presented here are consistent with those previously reported *in vitro*. Our observations highlight the importance of the size of amyloid particles and their associated membrane disruption abilities. A direct result of membrane disruption *in vivo* would lead to a loss in cell viability, which was confirmed by the CellTox Green assay (Promega) (Fig 3). This membrane integrity based assay distinguishes between intact viable cells and dead cells, and has been utilized to identify and characterize potential therapeutic agents in treating a parasitic disease whose mode of action is via altering cell membrane structure resulting in apoptosis [40]. Our results demonstrated that fragmented fibril samples caused a loss of plasma membrane integrity of SH-SY5Y cells, indicative of cytotoxicity. The cytotoxic effect was much greater than that caused by their longer unfragmented fibril counterparts. These results are in agreement with previous cell viability assays using MTT, in which fragmented fibril samples exhibited a higher cytotoxic effect in SH-SY5Y cells than unfragmented fibrils, suggesting a potential mode of action via membrane permeabilisation [17]. Furthermore, our CellTox data supports a potential mode of action by direct fibril-membrane interactions, causing a physical disruption of membranes and subsequent cell death. This would be an additional mechanism of action to that proposed by the internalization of the fragmented fibrils due to their reduced size [20]. It has also been demonstrated using liposomes and Cryo-EM that the fibril interaction is via the fibril end penetrating the lipid membrane and causing subsequent removal or blebbing [22]. Further to these observations our biophysical data, using TIRE analysis of natively derived cellular membranes, indicate a generic mechanism of membrane disruption by fibrils in which material is stripped (Fig 4), potentially by the blebbing mechanism observed by Cryo-EM [22].

Many proteins are capable of forming amyloid fibrils, including α-syn, Aβ_40_ and lysozyme, with each protein’s amyloid formation being associated with a disease-state: Parkinson’s disease, Alzheimer’s and hereditary systemic amyloidosis, respectively. Their interaction at cellular membranes is likely to be governed by electrostatic and hydrophobic interactions, as previously reported in the case of amyloid β, whose electrostatic interaction between charged peptide residues and lipid head groups modulated Aβ-membrane binding [41]. Furthermore, the hydrophobicity of fibril ends has been suggested as a common property that can cause dramatic distortions in lipid membranes [22]. This hydrophobicity could be responsible for a direct relationship between the number of fibrils ends, membrane binding and subsequent loss of material. This effect is elicited by fibrils formed from all three studied proteins, demonstrating an enhanced fibril-membrane interaction by fragmented species.

In conclusion, we provide additional mechanistic insight into how fibril samples interact with membranes by applying TIRE, showing that fragmented fibril samples cause significantly increased cytotoxicity by stripping material from plasma membranes.

## Supporting Information

S1 File Raw data associated with Figs 2, 3 and 4 can be found in the S1 File. (DOCX)
