# Supplementary material for: Analysis of Toxic Amyloid Fibril Interactions at Natively Derived Membranes by Ellipsometry
Source: PLoS One. 2015 Jul 14;10(7):e0132309. doi: 10.1371/journal.pone.0132309 (PMC4501548; doi:10.1371/journal.pone.0132309)
Supplement: S1 File — (DOCX) [file pone.0132309.s001.docx]

**Supplementary Data
Raw experimental data**

**Analysis of toxic amyloid fibril interactions at natively derived membranes by ellipsometry.**

Smith R.A.S.^1^ Nabok A.^2^ Blakeman B.J.F.^3^ Xue W-F.^3^ Abell B.M.^1^ Smith D.P.^1*^

1. Biomedical Research Centre, Sheffield Hallam University, Sheffield, United Kingdom.

2. Materials and Engineering Research Institute, Sheffield Hallam University, Sheffield, United Kingdom.

3. School of Biosciences, University of Kent, Canterbury, Kent, United Kingdom.

|  | **α-syn** | | **Lysozyme** | | **Aβ_40_** | |
| --- | --- | --- | --- | --- | --- | --- |
|  |  |  |  |  |  |  |
| rep | Unfragmented | Fragmented | Unfragmented | Fragmented | Unfragmented | Fragmented |
| 1 | 4030 | 6028 | 10974 | 12426 | 10496 | 12347 |
| 2 | 2738 | 4753 | 10726 | 13340 | 10278 | 17775 |
| 3 | 3267 | 5042 | 11535 | 22834 | 9957 | 11989 |
| 4 | 3079 | 3466 |  |  |  |  |
| 5 | 2407 | 4593 |  |  |  |  |
| 6 | 2860 | 4586 |  |  |  |  |
| ave | 3064 | 4745 | 11078 | 16200 | 10244 | 14037 |
| stdev | 558 | 827 | 414 | 5763 | 271 | 3242 |

**Fig 2 raw data. The effect of fibril fragmentation on length and its capacity to cause membrane disruption.** Liposome dye release assay using a model lipid membrane formed from 80% (w/w) phosphatidylcholine and 20% (w/w) phosphatidylserine encapsulating the fluorescent probe carboxyfluorescein. Dye-encapsulated vesicles were incubated with amyloid fibril samples (30 min), and fluorescence was recorded at that time point. Data was normalised relevant to the unfragmented sample for each of the proteins in question.

|  | **Full length** | |  |  |  | **Fragmented** | |  |
| --- | --- | --- | --- | --- | --- | --- | --- | --- |
| rep | 24h | 48h | 72h |  | rep | 24h | 48h | 72h |
| 1 | 10704 | 13615 | 18320 |  | 1 | 21458 | 46046 | 45737 |
| 2 | 9841 | 12794 | 20257 |  | 2 | 13929 | 40065 | 45390 |
| 3 | 9293 | 12514 | 18313 |  | 3 | 35174 | 40929 | 44740 |
| 4 | 8911 | 12769 | 34493 |  | 4 | 45861 | 42718 | 45778 |
| 5 | 11216 | 17504 | 51139 |  | 5 | 14553 | 64422 | 37930 |
| 6 | 11433 | 16782 | 24046 |  | 6 | 10730 | 40424 | 63382 |
| Blank | 7262 | 9380 | 11716 |  | Blank | 7262 | 9380 | 11716 |

**(A) α-syn**

**(B) Aβ_40_**

|  | **Full length** | |  |  |  | **Fragmented** | |  |
| --- | --- | --- | --- | --- | --- | --- | --- | --- |
| rep | 24h | 48h | 72h |  | rep | 24h | 48h | 72h |
| 1 | 8713 | 10381 | 13640 |  | 1 | 19726 | 29680 | 32695 |
| 2 | 8795 | 10666 | 13805 |  | 2 | 15184 | 32939 | 33845 |
| 3 | 9059 | 10887 | 13383 |  | 3 | 9928 | 28455 | 33755 |
| 4 | 8917 | 10872 | 13375 |  | 4 | 12087 | 31578 | 31461 |
| 5 | 8623 | 11031 | 14182 |  | 5 | 13264 | 33302 | 35842 |
| 6 | 9220 | 11452 | 14919 |  | 6 | 10721 | 29485 | 33695 |
| Blank | 8148 | 10819 | 14450 |  | Blank | 8148 | 10819 | 14450 |

**Fig 3 raw data. The effect of fragmented fibrils on cell viability by cell membrane integrity assay.** SH-SY5Y cells (2x10^4^/well) were plated with the addition of the CellTox dye and allowed to adhere. The unfragmented and fragmented fibril samples were then added (A) α-syn 7 µM and (B) Aβ_40_ 10 µM. Fluorescence was recorded as above at 24, 48 and 72 hours (520_Em_/ 485_Ex_). The data was averaged and the blank was then subtracted.

| **α-syn** | |  |  |  |  |  |  |  |  |  |  |  |
| --- | --- | --- | --- | --- | --- | --- | --- | --- | --- | --- | --- | --- |
|  |  |  |  |  |  |  |  |  |  |  |  |  |
|  | **Fragmented** | | |  |  |  |  | **Unfragmented** | | |  |  |
| µg/mL | Slide 1 | Slide 2 | Slide 3 | ave | stdev |  | µg/mL | Slide 1 | Slide 2 | Slide 3 | ave | stdev |
| 0 | 0 | 0 | 0 | 0.00 | 0.00 |  | 0 | 0 | 0 | 0 | 0.00 | 0.00 |
| 1 | -1.583 | -1.767 | -0.949 | -1.43 | 0.43 |  | 1 | -0.418 | -2.11 | -2.271 | -1.60 | 1.03 |
| 10 | -1.057 | -2.698 | -1.361 | -1.71 | 0.87 |  | 10 | -0.666 | -2.2745 | -1.376 | -1.44 | 0.81 |
| 100 | -0.884 | -2.899 | -1.9 | -1.89 | 1.01 |  | 100 | 1.043 | -1.034 | -2.221 | -0.74 | 1.65 |
| 250 | -1.152 | -3.443 | -2.513 | -2.37 | 1.15 |  | 250 | -0.143 | -1.215 | -1.122 | -0.83 | 0.59 |
| 500 | -3.485 | -4.211 | -6.611 | -4.77 | 1.64 |  | 500 | -1.348 | 0.348 | -0.938 | -0.65 | 0.88 |
|  |  |  |  |  |  |  |  |  |  |  |  |  |
| **Lysozyme** | |  |  |  |  |  |  |  |  |  |  |  |
|  |  |  |  |  |  |  |  |  |  |  |  |  |
|  | **Fragmented** | | |  |  |  |  | **Unfragmented** | | |  |  |
| µg/mL | Slide 1 | Slide 2 | Slide 3 | ave | stdev |  | µg/mL | Slide 1 | Slide 2 | Slide 3 | ave | stdev |
| 0 | 0 | 0 | 0 | 0.00 | 0.00 |  | 0 | 0 | 0 | 0 | 0.00 | 0.00 |
| 1 | 0.055 | 0.049 | 0.244 | 0.12 | 0.11 |  | 1 | -0.506 | -0.433 | 0.044 | -0.30 | 0.30 |
| 5 | 0.431 | 0.813 | 0.315 | 0.52 | 0.26 |  | 5 | -0.148 | -0.076 | 0.362 | 0.05 | 0.28 |
| 10 | 0.585 | 0.835 | 1.31 | 0.91 | 0.37 |  | 10 | -0.092 | 0.206 | -0.32 | -0.07 | 0.26 |
| 15 | -0.408 | -0.193 | -0.814 | -0.47 | 0.32 |  | 15 | -0.137 | -0.102 | -0.648 | -0.30 | 0.31 |
| 20 | -0.585 | -0.593 | -0.733 | -0.64 | 0.08 |  | 20 | -0.178 | -0.136 | -0.024 | -0.11 | 0.08 |
| 25 | -1.091 | -0.203 | -0.171 | -0.49 | 0.52 |  | 25 | -0.203 | -0.107 | -0.1 | -0.14 | 0.06 |
| 35 | -0.223 | -1.576 | -0.622 | -0.81 | 0.70 |  | 35 | -0.055 | -0.044 | -0.602 | -0.23 | 0.32 |
| 50 | -0.952 | -0.06 | -0.824 | -0.61 | 0.48 |  | 50 | -0.173 | -0.183 | 0.02 | -0.11 | 0.11 |
|  |  |  |  |  |  |  |  |  |  |  |  |  |
|  |  |  |  |  |  |  |  |  |  |  |  |  |
| **Aβ_40_** |  |  |  |  |  |  |  |  |  |  |  |  |
|  |  |  |  |  |  |  |  |  |  |  |  |  |
|  | **Fragmented** | | |  |  |  |  | **Unfragmented** | | |  |  |
| µg/mL | Slide 1 | Slide 2 | Slide 3 | ave | stdev |  | µg/mL | Slide 1 | Slide 2 | Slide 3 | ave | stdev |
| 0 | 0 | 0 | 0 | 0.00 | 0.00 |  | 0 | 0 | 0 | 0 | 0.00 | 0.00 |
| 1 | -0.697 | -0.091 | -0.247 | -0.34 | 0.31 |  | 1 | -0.037 | 0.165 | -0.03 | 0.03 | 0.11 |
| 5 | -0.24 | -0.05 | -0.337 | -0.21 | 0.15 |  | 5 | -0.257 | -0.169 | -0.058 | -0.16 | 0.10 |
| 10 | 0.294 | -0.051 |  | -0.40 | 0.92 |  | 10 | -0.083 | -0.109 | 0.123 | -0.02 | 0.13 |
| 15 | -0.217 | -0.262 | -0.064 | -0.18 | 0.10 |  | 15 | -0.385 | -0.113 |  | -0.44 | 0.36 |
| 20 | -0.533 | -0.351 | -1.266 | -0.72 | 0.48 |  | 20 | -0.292 | -0.316 | -0.079 | -0.23 | 0.13 |
| 25 | -1.041 | -0.216 | -0.022 | -0.43 | 0.54 |  | 25 | -0.255 | -0.309 | -0.23 | -0.26 | 0.04 |
| 35 | -0.216 | -0.324 | -0.156 | -0.23 | 0.09 |  | 35 | -0.267 | -0.349 | -0.496 | -0.37 | 0.12 |
| 50 | -0.355 | -0.3 | -0.302 | -0.32 | 0.03 |  | 50 | -0.239 | -0.093 | -0.135 | -0.16 | 0.08 |

**Fig 4 raw data. TIRE measurements of membrane disruption at native lipid membrane by the incremental addition of amyloid fibrils.** Natively derived cells from the neuronal cell line; SH-SY5Y was deposited by Langmuir-Schaefer deposition and protein: lipid interactions were probed between amyloid fibril samples and plasma membranes. Fibril samples: unfragmented (left); and fragmented (right). (A+D) α-syn, (B+E) Lysozyme and (C+F) Aβ_40_. Membrane thickness measured by change in Δd (nm) versus fibril concentration. (n=3, SE). Two data points were removed due to the injection of an air bubble at this sample point making the data unusable.
